# Perceived stress and depressive symptoms among Chinese college students: A moderated mediation model of biorhythm and ego resilience

**DOI:** 10.3389/fpubh.2022.951717

**Published:** 2022-08-04

**Authors:** Yao Ma, Baiyang Zhang, Yajing Meng, Yuan Cao, Yineng Mao, Changjian Qiu

**Affiliations:** ^1^Mental Health Center, West China Hospital of Sichuan University, Chengdu, China; ^2^West China School of Public Health and West China Fourth Hospital, Sichuan University, Chengdu, China; ^3^Department of Nuclear Medicine, West China Hospital of Sichuan University, Chengdu, China; ^4^Huaxi MR Research Center (HMRRC), Department of Radiology, West China Hospital of Sichuan University, Chengdu, China; ^5^School of Public Health, North Sichuan Medical College, Nanchong, China

**Keywords:** depression, college students, adolescents, biorhythm, perceived stress, moderating mediation, resilience

## Abstract

**Objective:**

To explore whether biological rhythm disturbance mediates the association between perceived stress and depressive symptoms and to investigate whether ego resilience moderates the mediation model.

**Methods:**

A cross-sectional study was carried out using an online self-report questionnaire distributed to college students from September 2021 to October 2021. Patient Health Questionnaire-9 (PHQ-9), Perceived Stress Severity (PSS-10), the Biological Rhythms Assessment in Neuropsychiatry (BRIAN), and Ego Resilience (ER-96) were used for investigation. SPSS 23 was used for data analyses. The significance of mediation was determined by the PROCESS macro using a bootstrap approach.

**Results:**

Among the participants, 9.2% (*N* = 1,282) exhibited significant symptoms of depression. Perceived stress was positively associated with depressive symptoms, and biorhythm partially mediated this relationship. The direct and indirect effects were both moderated by ego resilience. Perceived stress had a greater impact on depressive symptoms and biorhythm for college students with lower ego resilience, and the impact of biorhythm on depressive symptoms was also stronger for those with lower ego resilience. Perceived stress had an impact on depressive symptoms directly and indirectly via the mediation of biorhythm.

**Conclusion:**

Schools and educators should guide college students to identify stress correctly and provide effective suggestions to deal with it. Meanwhile, maintaining a stable biorhythm can protect college students from developing depressive symptoms. Students with low resilience should be given more attention and assistance.

## Introduction

Depression is characterized by persistent pessimism and lack of interest or pleasure and is considered one of the most frequent mental disorders among college students (1). Several cross-sectional studies reported that the prevalence of depressive symptoms among Chinese college students ranged from 12.2 to 28.9% (2–4). The college years are distinct periods of development straddling the adolescent and young adulthood stage, as well as one of the common times for the onset of depression (5, 6). Since depression at an early age has negative consequences in long-term life (7), the current situation and factors related to depressive symptoms among college students are worth exploring.

Depression is generally considered a stress-related disorder. Statistically, nearly 70% of primary depression is caused by stress (8). Stress perception refers to a person's appraisal of the intensity of threat from stressors. Severe life stressors impact the risk of depression (9, 10). College students are confronted with considerable stress, including environmental changes, academic requirements, higher education programs and the transition to independence (11), and are therefore a vulnerable group in need of attention. A 1-year follow-up among college students found that stressful experiences are one of the strongest predictors of depression (12), but how perceived stress exerts its effects on depressive symptoms remains unclear. Some studies have reported that college students with higher perceived stress suffer from more serious sleeping problems (13, 14) and report lower physical activity and longer sedentary time (15). Additionally, perceived stress was generally accompanied by dietary behavior changes (16).

Biorhythm refers to the cyclical changes of various functional activities of an individual to maintain the homeostasis of the internal environment and adapt to the changes of the external environment. Sleeping, daily activities, eating patterns and social interactions are uniformly defined as behavioral markers of biorhythm, which often present with abnormalities in depressive individuals (17, 18). A meta-analysis indicated an association between biorhythm orientation and more severe depressive symptoms (19). At present, the most commonly evaluated is chronotype, the diurnal preference for the sleep-wake circle and daily activity, which lies on a continuum between morningness and eveningness (20). Surveys on college students found higher depressive symptom severity in non-morning chronotypes and moderate/severe perceived stress groups (21–25). Weight, appetite changes and loss of energy are also extremely common in adolescents with depressive symptoms in addition to changes in sleep and activity levels (26), but there is a lack of a multidimensional evaluation of their associated biorhythms. In this study, we will comprehensively evaluate the biorhythm of college students through a scale specially designed to evaluate biorhythm, and explore the role in depressive symptoms. Since college students have not yet established a stable life structure, comprehensively characterizing the biorhythm among college students will help in understanding the effects of biorhythm disturbance on depression and provide reliable evidence for preventive interventions. It is recognized that the stress response causes neurohumoral turbulence in the regulation of the biorhythm and further leads to mental disorders (27). Therefore, we hypothesized that biorhythm disturbances might play a mediating role in the progression of perceived stress into depressive symptoms. Liu et al. identified the mediating role of insomnia in perceived stress and depressive symptoms among Chinese college students (28), but other aspects concerning biorhythm changes were not discussed. A more comprehensive investigation of biorhythm and its potential mediating role in the relationship between perceived stress and depressive symptoms among college students deserves further exploration.

Resilience is the adjustment and adaptive ability of an individual to changes in the external environment in psychology and behavior (29). As a relatively stable psychological trait, resilience varies among individuals, serving as a vital protective factor in psychological health (30, 31). High resilience can protect college students from depressive symptoms (32). Moreover, resilience plays an important role in the regulation of stress (33, 34). Physiologically, high resilience can alleviate neurohormonal changes in the body and have a positive impact on biorhythm stability (35). Therefore, we conjectured that high ego resilience attenuated the effects of perceived stress on depressive symptoms and further explored the moderating effect of resilience on the mediation model.

**Figure 1:**
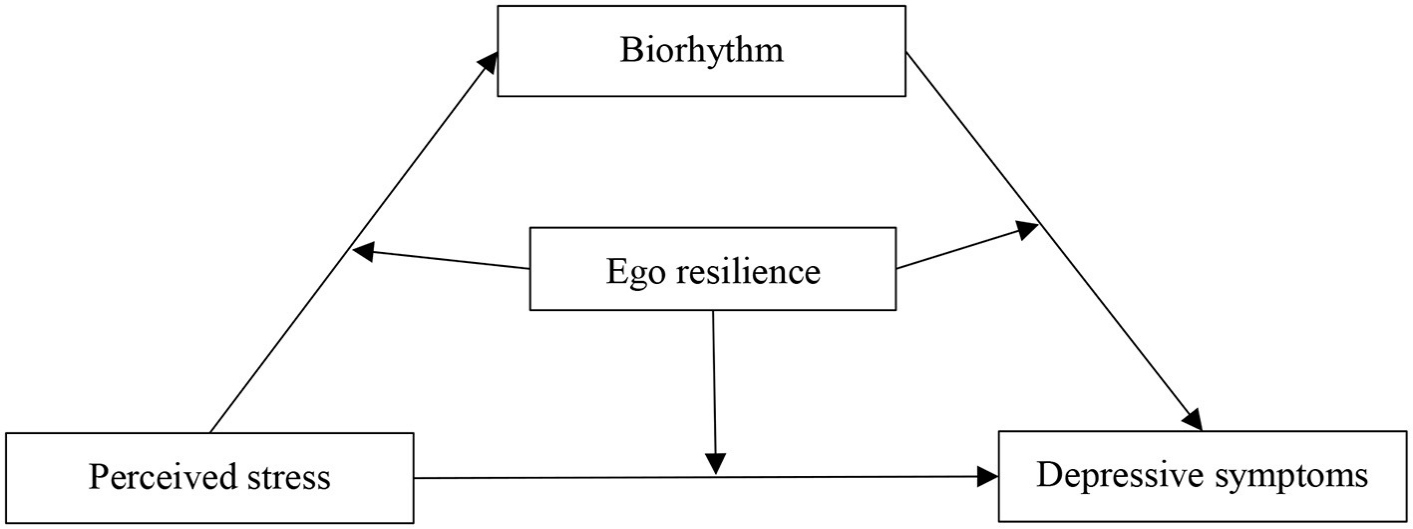
Conceptual moderating mediation model.

In summary, this study aimed to explore the interrelationships among perceived stress, biorhythm, ego resilience and depressive symptoms and to test the hypothetical moderated mediation model (Figure 1), hoping to provide a reference for targeted interventions to improve the psychological health of college students.

## Methods

### Participants and procedures

An online investigation with convenience sampling was conducted from September 2021 to October 2021. Our questionnaires were in the local language and all scales are Chinese versions translated by previous scholars. The inclusion criteria of participants were college students in China. Data were collected via the Wenjuanxing platform (https://www.wjx.cn/vj/hP4DYhi.aspx). Basic questions are set in the questionnaire to ensure that the questionnaires submitted normally are from college students. Missing any questions will fail to submit the questionnaire properly. In data preprocessing, we also manually excluded some obviously sloppy answers (i.e., the age is obviously beyond the range of normal college students, the IP address is from the same person, and too little time to answer questions, which suggested that the questionnaire was completed casually). This study was approved by the Ethics Committee of West China Hospital of Sichuan University. All participants were informed with online consent. In total, 15,879 questionnaires were received, and a final sample of 13,943 (effective response rate: 87.81%) was analyzed after the elimination of the unqualified.

### Measures

#### Basic information

Information concerning age, gender, and grade was collected to recognize the demographic characteristics of the participants.

#### Independent variable: Perceived stress

Stress levels were measured by the Perceived Stress Scale (PSS), which contains 10 items rated on a 5-point scale from 0 (never) to 4 (very often) (36). Participants reported the degree to which situations in one's life have been unpredictable, uncontrollable and overloaded over the last month, and higher scores represent higher stress perception. The total score ranges from 0 to 40, with higher scores indicating greater perceived stress. In this study, Cronbach's α of the scale is 0.883.

#### Mediator: Biorhythm

Biorhythm was assessed by the Biological Rhythms Interview of Assessment in Neuropsychiatry (BRIAN), a screening measure assessing the biorhythm of activity, sleeping, eating and socializing (37). Each item is scored from 1 to 5, with a total score ranging from 18 to 90, and higher scores suggest more disturbed biorhythms. Cronbach's α of the entire scale is 0.936 in the current study.

#### Moderator: Ego resilience

Ego resiliency was measured by the ego-resiliency scale (ER-96), which consists of 14 items (29). Questions solicited responses on a scale ranging from 1 (does not apply at all) to 4 (applies very strongly), and higher scores represent stronger ego resilience. In the current study, Cronbach's α of the scale was 0.909.

#### Dependent variable: Depressive symptoms

Depressive symptoms were measured by PHQ-9, a brief self-administered instrument, and scores each of the items as “0” (not at all) to “3” (nearly every day) (38). The most common scoring criteria are 0–4: no depression, 5–9: probably mildly depression, 10–14: probably moderately depression, 15–19: probably moderately severe depression, and 20–27: probably severely depression. A cutoff score of 10 or higher is generally accepted as a current depressive episode, which was consistent with moderate and severe severity of depression (39). Cronbach's α of the entire scale was 0.943 in the current study.

### Statistical analysis

SPSS 23 for Windows was applied for statistical analysis. Descriptive statistics (mean ± standard deviation were used for quantitative variables, while the constituent ratio for categorical variables) were used to describe the participants' demographic characteristics and the main study variables (perceived stress, biorhythm, ego resilience and depressive symptoms). One-way analyses of variance (ANOVAs) and *t*-tests were used to make a statistical comparison. Pearson's rho correlations were computed to examine the association between the variables.

To test our hypothesis concerning the direct and indirect effects of perceived stress on depressive symptoms, the following conditions are required: The independent variable predicts the mediator variable and the dependent variable and the mediator variable predicts the dependent variable; after the addition of mediating variable, the impact of the independent variable on the dependent variable turns into weaker (partial mediation) or non-significant (full mediation). We used serial multiple mediator models with the PROCESS macro for SPSS described by Hayes (40), which can provide the significance of the regression coefficients for each path, and estimate the size of the mediation effects. Model 4 was performed to identify the mediating effects. We performed 5,000 bootstrap resamples to determine the 95% confidence interval (CI) of the indirect effect of perceived stress on depressive symptoms. After confirming the mediation effect of biorhythm was significant, model 59, a moderated mediation model, was used to estimate the moderating role of ego resilience. There is a significant mediating effect if the 95% CI excludes 0. The significance level is set at 0.05 for all tests.

## Results

### Common method biases test

The Harman single-factor test was used to ensure the reliability and accuracy of the data since we used online self-reports. The results showed that there were seven factors with eigenvalues >1, and the variance explained by the first factor was 27.27%, less than the critical standard of 40%. In summary, there was no significant common method deviation in this study.

### Comparison of perceived stress, biorhythm, depressive symptoms and mental toughness among college students with different sociodemographic characteristics

Among the eligible samples in this study, 5,057 (36.3%) were male and 8,886 (63.7%) were female. The mean age of the sample was 19.14 (SD = 1.22). As for the severity of depressive symptoms, 9,555 (68.5%) students were normal, 3,106 (22.3%) presented mild depressive symptoms, 788 (5.7%) presented moderate depressive symptoms, 335 (2.4%) presented moderately severe symptoms, 159 (1.1%) presented severe depressive symptoms. In total, the detection rate of depression was 9.2% (*n* = 1,282) at a threshold of 10, which included moderate and severe severity of depressive symptoms.

Female reported significantly higher scores for perceived stress (male = 13.61 ± 7.99, female = 14.98 ± 6.24, *p* < 0.001), ego resilience (men = 39.25 ± 11.16, women = 39.81 ± 8.05, *p* < 0.001) and depressive symptoms (male = 3.44 ± 4.75, female = 3.78 ± 4.50, *p* < 0.001), while there was no significant difference in biorhythm (*p* = 0.99) between genders.

In addition, age was positively correlated with depressive symptoms (*r* = 0.28, *p* = 0.01) and biorhythm (*r* = 0.35, *p* < 0.001) and negatively correlated with resilience (*r* = 0.049, *p* < 0.001). Age was not statistically related to perceived stress (*p* = 0.234).

The Pearson's correlations for stress perception, biorhythm and depressive symptoms are presented in Table 1. The results showed that depressive symptoms had a positive correlation with perceived stress (*r* = 0.417, *p* < 0.001) and biorhythm (*r* = 0.608, *p* < 0.001). Perceived stress was also positively correlated with biorhythm (*r* = 0.516, *p* < 0.001).

**Table 1:** Correlations (*r*) between perceived stress, biorhythm, ego resilience and depressive symptoms (*n* = 13,943).

	**Stress perception**	**Biorhythm**	**Ego resilience**	**Depressive symptoms**
Stress perception	–	0.516**	0.254**	0.417**
Biorhythm	0.516**	–	−0.031**	0.608**
Ego resilience	0.254**	−0.031	–	−0.147**
Depression symptom	0.417**	0.608**	−0.147**	–
***M*** ± SD	14.48 ± 6.960	30.68 ± 10.039	39.61 ± 9.301	3.65 ± 4.594

**p < 0.001.

### Mediation effect analysis

**Figure 2:**
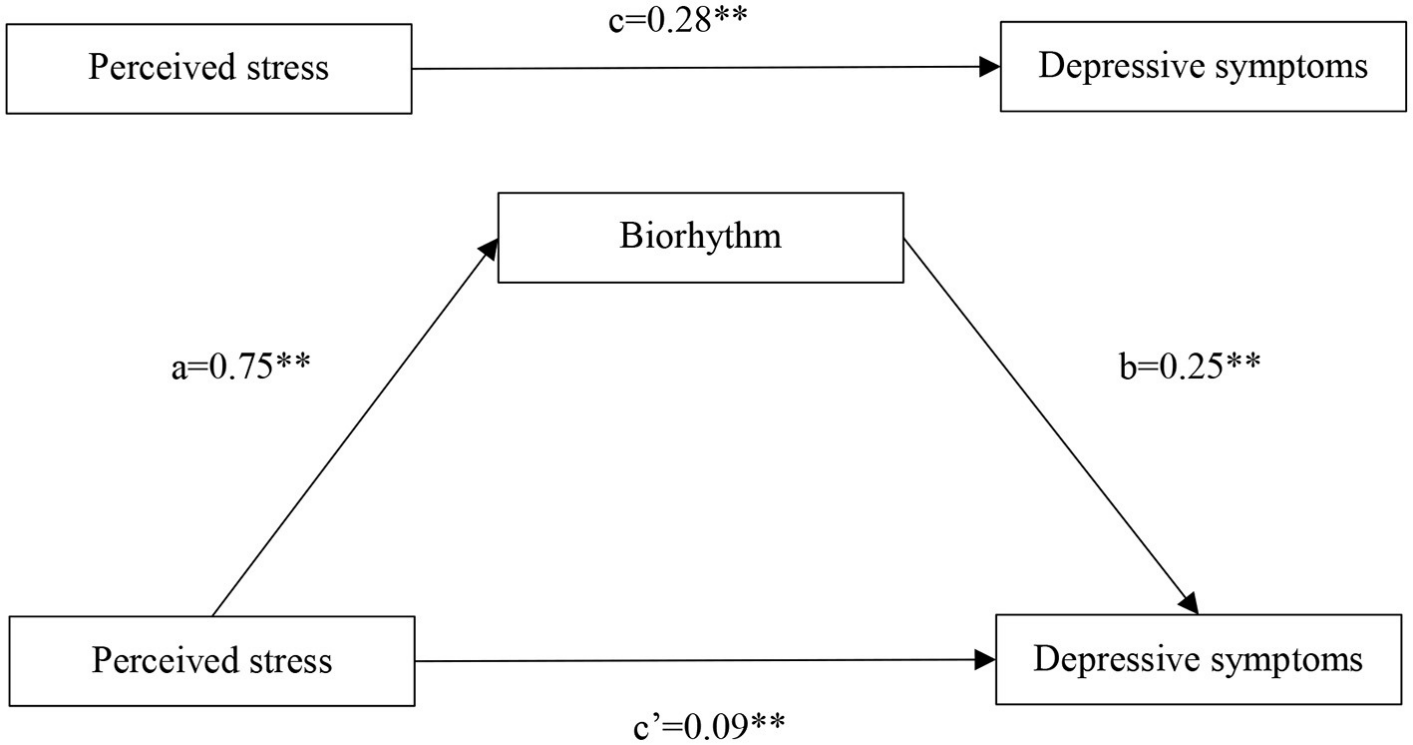
Propose models that investigate mediated effects (**p < 0.001).

The results of the bootstrapping methods showed that the direct, indirect and total effects in the mediation model were significant after controlling for age and gender (Table 2). Perceived stress was positively associated with depression (β = 0.28, *p* < 0.001). Perceived stress was significantly positively associated with biorhythm (β = 0.75, *p* < 0.001). When putting perceived stress and biorhythm into the regression equation, both perceived stress (β = 0.09, *p* < 0.001) and biorhythm (β = 0.25, *p* < 0.001) were significantly positively associated with depressive symptoms in college students. The bootstrap confidence interval of the mediation effect did not include 0 as was shown in Table 3, showing that biorhythm had a significant mediating effect between perceived stress and depressive symptoms, and the mediating effect accounted for 66.7% of the total effects. Figure 2 illustrates the mediation model, along with the standardized path coefficients.

**Table 2:** Mediating effect of biorhythm between perceived stress and depressive symptoms.

**Predictors (IV)**	**Model 1 (perceived stress**→**depressive symptoms)**		**Model 2 (perceived stress**→**biorhythm)**		**Model 3 (perceived stress, biorhythm**→**depressive symptoms)**	
	***B***	***t***	***B***	***t***	***B***	***t***
Gender	−0.03	−0.36	−0.998	−6.57**	0.22	3.40**
Age	0.12	4.13**	0.31	5.24**	0.04	1.72
Perceived stress	0.28	54.04**	0.75	71.67**	0.09	17.75**
Biorhythm	–	–	–	–	0.25	68.85**
*R* ^2^	0.18		0.27		0.38	
*F*	985.48**		1,720.0**		2,175.38**	

**p < 0.001.

**Table 3:** Mediating model examination by bootstrap.

	**Perceived stress**→**depression**			
	**Effect**	**SE**	**LL 95%CI**	**UL 95%CI**
Direct effect	0.092	0.005	0.081	0.102
Indirect effect	0.184	0.005	0.175	0.193
Total effect	0.276	0.005	0.266	0.286

### Moderation effect analysis

**Figure 3:**
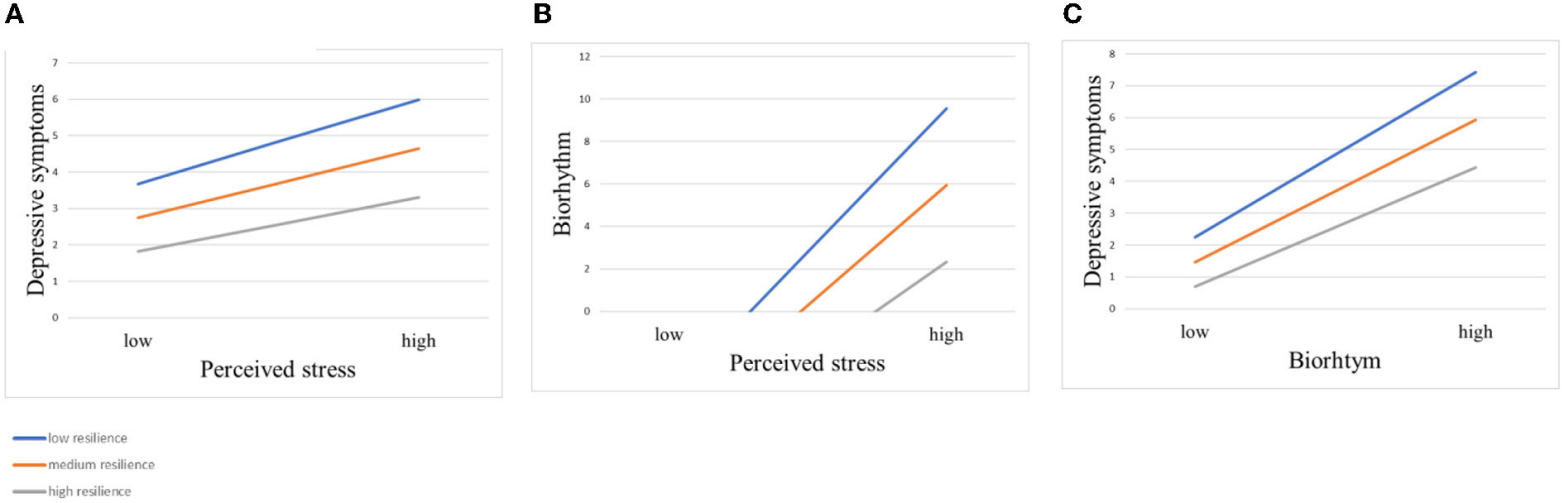
Moderating effects of different levels of resilience on the relationship between perceived stress and depressive symptoms. **(A)** Moderating effect of different levels of resilience on perceived stress and depressive symptoms. **(B)** Moderating effect of different levels of resilience on perceived stress and biorhythm. **(C)** Moderating effect of different levels of resilience on biorhythm and depressive symptoms.

Table 4 shows the results of the moderating mediation. First, resilience directly moderates the relationship between perceived stress and depressive symptoms. When ego resilience was low (−1 SD), perceived stress had a greater impact on depressive symptoms (effect = 0.166, *p* < 0.001), with a 95% confidence interval [0.152, 0.180]. When the individual's ego resilience was high (+1 SD), perceived stress had little effect on depressive symptoms (effect = 0.107, *p* < 0.001), and the 95% confidence interval was [0.095, 0.120]. In addition, ego resilience moderated both the anterior and posterior pathways of the mediation effects. The effects of perceived stress on biorhythm and biorhythm on depressive symptoms were greater when resilience was lower. To analyze the moderating effect of resilience more intuitively, the participants were divided into a high group (those whose resilience score was one standard deviation higher than the mean), a middle group (those whose resilience score was equal to the mean) and a low group (those whose resilience score was one standard deviation lower than the mean). The moderating effect charts were drawn stratified by resilience score group. Figure 3 shows that in the low resilience group, biorhythm scores and depressive symptoms scores increased rapidly with an increase in perceived stress scores. However, in the high resilience group, the increasing slope of the biorhythm score and depressive symptom score was relatively gentle.

**Table 4:** Moderated mediation analysis.

	**Biorhythm**			**Depressive symptoms**		
	***B***	**SE**	***t***	***B***	**SE**	***t***
Gender	−1.36	0.15	−9.16**	0.07	0.06	1.13
Age	0.25	0.06	4.34**	0.02	0.02	0.82
Perceived stress	0.81	0.01	77.50**	0.14	<0.01	25.83**
Ego resilience	−0.27	<0.01	−30.30**	−0.12	<0.01	−31.76**
Perceived stress × ego resilience	−0.02	<0.01	−19.86**	−0.003	<0.001	−6.75**
Biothythm × ego resilience	–	–	–	−0.004	<0.001	−9.92**
*R* ^2^	0.32			0.43		
*F*	1,293.75**			1,503.09**		

B, coefficient; SE, stand error.

**p < 0.001.

## Discussion

These results suggest a positive association between perceived stress and depressive symptoms, which was mediated by biorhythm. In addition, resilience moderated the direct and indirect effects of the mediation model, serving as an internal resource in college students' ability to deal with perceived stress and reducing their risk of developing depressive symptoms.

### Depressive symptoms in college students

In the present study, the proportion of college students who were suffering from depressive symptoms was 9.2%, which was lower than that of other studies among Chinese college students (2–4). Regional differences, periods, and varied measurements may explain some discrepancies among studies. Most of our samples came from Sichuan Province, and the investigation period was during the normal period of Corona Virus Disease 2019, therefore the stress perception intensity may generally be lower than that during the outbreak period. In addition, there are differences in sensitivity and specificity for depressive symptoms in different self-rating scales of depressive symptoms. Female students reported higher stress perception and more severe depressive symptoms, which is consistent with previous studies (41–43). Men possess greater reactivity to the environment and better-coping abilities (44). Interestingly, female students were more resilient than men despite showing more severe depressive symptoms. The reason may be that resilience has different moderating abilities to different stressors and cannot buffer the effect against some stressors, such as the physical and emotional neglect experienced by women (45). Future studies can further identify gender differences in the response to different types of stress to clarify the regulatory mechanism.

Our study also found that younger college students displayed more severe depressive symptoms. Younger college students are more likely to be freshmen, who are at increased risk of self-reported severe depression (42), although they have the lightest class load compared to students in other grades. Sudden environmental changes from relatively regular high school life to the unfamiliar college life may be the explanation, since secondary school life is dominated by specific lectures, while college life requires more self-management to meet academic requirements and more diversified social skills. With the advances in age and social experience, coping strategies for different kinds of stressful events and emotion regulation abilities are improved.

### Direct impact of perceived stress on depressive symptoms

Along with previous findings, the present study revealed a positive association between perceived stress and depressive symptoms. The stress response is divergent in college students. Contributing factors consist of the nature of the events, behavioral and genetic factors, previous experience and personal resources. Severe stress perception leads to a range of emotional responses when individuals feel inadequate to cope with challenges. Chronic stress increases the severity of depressive symptoms (6, 46). Stress also induces serotonin alterations, which disrupts mood regulation and serves as critical pathogenesis of depression (47). Moreover, psychological stress could suppress the immune system (48), leading to changes in immune cytokine levels that may generate depressive symptoms (49).

### Mediating role of biorhythm

Our findings corroborate our hypothesis that biorhythm may be a mediator in the association between perceived stress and depressive symptoms. When stress is perceived, the human body needs to maintain relative homeostasis through a series of physiological and biochemical reactions (50). The stress response plays a causal role in depression onset, perhaps via neurobiological hyperactivation of the hypothalamic-pituitary–adrenal (HPA) axis and sympathetic nervous system (SNS) (51, 52). Typical responses to acute stress, such as immediate threats to physiological homeostasis, activate the SNS, with the release of catecholamines, contributing to the biorhythm (53, 54). Alternatively, activation of the HPA axis is crucial in the response to psychological stress and dysfunction of the HPA axis plays a crucial role in the development of depression (55). Normally, hormones controlled by the HPA axis fluctuate regularly throughout the day and display biorhythm involving sleeping, digestion, and activity (56).

First, sleep disturbance is a defining feature of depression, presenting as difficulty in sleep onset, mid-nocturnal, and early morning insomnia symptoms (57). It is considered not only a common finding of depression but also a significant predictor of the onset of depression (58, 59). Sleep disturbance disrupts stability and regulation (60) and hence may play a role in the pathophysiology of depressive symptoms.

Second, low activity levels are associated with subsequent depressive symptoms (61, 62). Patients with depression perform less physical activity (17, 63). A large-scale genome-wide association study (GWAS) also verified the predictive effect of physical activity on depression (64). Furthermore, stress alters ghrelin levels, which are involved in the regulation of appetite and digestion (65). Diet-induced changes in the gastrointestinal microbiome can affect mood and behavior by altering the gut microbiome (66). Weight change in depressed people may be a result of an increase or decrease in appetite (67).

Social function, defined as the ability to perform regular social roles, is considered a nonnegligible sign of depression (68). Social deficits are also manifestations of a state of depression (69). The interpersonal difficulties might be a result of reduced motivation (70). Depressed subjects rated life stimuli as more negative and arousing, which is associated with reduced social and emotional competence (71). In summary, our results demonstrated the important roles of these aspects involved in biorhythm changes in the development of depressive symptoms.

### Moderating role of ego resilience

Our results revealed that ego resilience may function as a moderator between perceived stress and depressive symptoms separately, as well as in the direct and indirect effects. Resilience not only moderates the relationship between perceived stress and depressive symptoms but also the association between perceived stress and biorhythm, as well as the relationship between biorhythm and depressive symptoms. Specifically, compared to high ego resilience individuals, the direct and indirect effects of perceived stress on depressive symptoms are greater in individuals with low ego resilience. That is, resilience can mitigate the negative effects of perceived stress. As an important psychological trait, resilience serves as a restorative factor to resist negative stimuli and adverse situations. The reason may be that people with high ego resilience usually have more social support and more positive emotional regulation (72, 73) and hence can stabilize their biorhythm and show milder depressive symptoms. The upper limit of stress tolerance of resilience may affect the risk of depression in college students under stress: strong resilience can help individuals adapt well, while poor resilience leads to failure to properly handle stress. College students with lower ego resilience should be taken more seriously when they perceive stress.

Our findings suggest that biorhythm is a critical mediator in the development of depressive symptoms, providing reasonable suggestions for reducing the incidence of depression among college students from the perspective of biorhythm regulation and coping strategies when reacting to stressors. Proper campus life arrangements, such as providing sports activities, social activities, regular daily schedules and the popularization of mental health knowledge, can help alleviate the consequences of severe stress perception.

## Conclusion

Perceived stress has an impact on depressive symptoms directly and indirectly via the mediation of biorhythm. Ego resilience moderates the association between perceived stress and depressive symptoms, as well as the relationships between perceived stress and biorhythm and between biorhythm and depressive symptoms.

Schools and educators should guide college students to identify stress correctly and provide effective suggestions to deal with it. Meanwhile, maintaining a stable biorhythm can protect college students from developing depression when perceiving stress, thus achieving the goal of reducing depressive symptoms. Students with low resilience should be given more attention and assistance.

### Limitations

There are some limitations to this study as this is an online study. First, our results found a higher proportion of females and a higher detection rate of depressive symptoms in females, thus possibly overestimating the detection rate of depressive symptoms in the entire college population. Future studies can use more theoretical sampling methods to investigate the prevalence of depressive symptoms among college students. Second, we adopted a self-report survey method, so there may be recall bias. Third, the psychological impact of major public health events on the population cannot be excluded. In addition, causal inference cannot be established from this cross-sectional study, and a large-scale prospective study is needed to further assess the critical role of biorhythm in depressive symptoms.

## Data availability statement

The original contributions presented in the study are included in the article/supplementary material, further inquiries can be directed to the corresponding author.

## Ethics statement

The studies involving human participants were reviewed and approved by Ethics Committee of West China Hospital of Sichuan University. The patients/participants provided their written informed consent to participate in this study.

## Author contributions

YMa and CQ: conceptualization. YMa and BZ: methodology. YMa: software and formal analysis and writing—original draft preparation. YMa, YMeng, YC, and YMao: investigation. YMa, CQ, BZ, YMeng, and YC: writing—review and editing. All authors contributed to the article and approved the submitted version.

## Funding

This study was funded by the Department of Science and Technology of Sichuan provincial government (Grant No. 2022YFS0345).

## Conflict of interest

The authors declare that the research was conducted in the absence of any commercial or financial relationships that could be construed as a potential conflict of interest.

## Publisher's note

All claims expressed in this article are solely those of the authors and do not necessarily represent those of their affiliated organizations, or those of the publisher, the editors and the reviewers. Any product that may be evaluated in this article, or claim that may be made by its manufacturer, is not guaranteed or endorsed by the publisher.
